# Neuropsychological determinants of the cognitive response to combined physical and cognitive training in adults with mild cognitive impairment

**DOI:** 10.1002/alz70857_102937

**Published:** 2025-12-25

**Authors:** Christine Gagnon, Manuel Montero‐Odasso, Guangyong Zou, Mark R. Speechley, Quincy J Almeida, Teresa Liu‐Ambrose, Laura E. Middleton, Richard Camicioli, Nick W Bray, Karen Li, Sarah Fraser, Frederico Pieruccini‐Faria, Amer M. Burhan, Nicolas Berryman, Maxime Lussier, Surim Son, J Kevin Shoemaker, Louis Bherer

**Affiliations:** ^1^ Montreal Heart Institute, Montreal, QC, Canada; ^2^ Schulich School of Medicine & Dentistry, Division of Geriatric Medicine, Western University, London, ON, Canada; ^3^ Parkwood Institute, London, ON, Canada; ^4^ Schulich School of Medicine & Dentistry, Western University, London, ON, Canada; ^5^ University of Wester Ontario, London, ON, Canada; ^6^ Wilfrid Laurier University, Waterloo, ON, Canada; ^7^ Vancouver Coastal Health Research Institute, Vancouver, BC, Canada; ^8^ Centre for Aging SMART, Vancouver Coastal Health Research Institute, Vancouver, BC, Canada; ^9^ University of British Columbia, Vancouver, BC, Canada; ^10^ Djavad Mowafaghian Centre for Brain Health, Vancouver, BC, Canada; ^11^ Schlegel‐UW Research Institute for Aging, Waterloo, ON, Canada; ^12^ University of Waterloo, Waterloo, ON, Canada; ^13^ University of Alberta, Edmonton, AB, Canada; ^14^ University of Western Ontario, London, ON, Canada; ^15^ Concordia University, Montreal, QC, Canada; ^16^ University of Ottawa, Ottawa, ON, Canada; ^17^ Gait and Brain Laboratory, Parkwood Institute, London, ON, Canada; ^18^ Gait & Brain Lab; Lawson Research Institute; Schulich School of Medicine& Dentistry, Division of Geriatric Medicine, Western University, London, ON, Canada; ^19^ University of Toronto, Toronto, ON, Canada; ^20^ Parkwood Institute‐Mental Health, Western University, London, ON, Canada; ^21^ Western University, London, ON, Canada; ^22^ Ontario Shores Centre for Mental Health Sciences, Whitby, ON, Canada; ^23^ Université du Québec à Montréal, Montréal, QC, Canada; ^24^ University of Montreal, Montreal, QC, Canada; ^25^ Centre de Recherche de l'Institut Universitaire de Gériatrie de Montréal (CRIUGM), Montreal, QC, Canada; ^26^ Université de Montréal, Montréal, QC, Canada; ^27^ Centre de Recherche de l'Institut Universitaire de Gériatrie de Montréal, Montréal, QC, Canada; ^28^ Montreal Heart Institute, Montréal, QC, Canada

## Abstract

**Background:**

In recent years, many studies have investigated the effects of non‐pharmacological interventions (e.g. physical and cognitive training) on cognition in patients with mild cognitive impairment. The SYNERGIC trial showed that combining physical exercise and cognitive training leads to greater gains on the ADAS‐Cog than an active control condition, and physical exercise alone. Yet not all participants showed the same cognitive gains. To date, little is known about the cognitive characteristics of *responders* vs *non‐responders* to non‐pharmacological interventions.

**Objective:**

To compare baseline cognitive profiles of *responders* and *non‐responders* to physical exercise training alone or in combination with cognitive training.

**Methods:**

Of the 175 initially randomized individuals with MCI in the SYNERGIC trial, 143 completed the 6‐month assessment. Regardless of randomization, participants were identified as *responders* if the change in ADAS‐Cog‐13 (T6‐T0) was negative, indicating improved performances, and *non‐responders* were determined if change was null or positive, indicating decreased performance. Composite z‐scores were calculated from the baseline neuropsychological assessment: Global cognition (MoCA), Language (Boston Naming Test, Semantic Verbal fluency), Memory (MoCA delayed recall subscore), Processing Speed (TMT A, Stroop Naming, Stroop Reading), Working Memory (Digit span forward and backward), and Executive Functions (TMT B, Stroop Inhibition and Switching).

**Results:**

*Responders* (*n* = 91; 72yrs, 46% female, ADAS‐Cog(T0): 15.81) and *non‐responders* (*n* = 52; 74.2yrs, 54% female, ADAS‐Cog(T0): 15.13) were comparable for sex, baseline ADAS‐Cog; a trend was observed for older age in non‐responders (*p* = .062). Overall, at baseline, *responders* had better global cognition (0.14±0.94 vs ‐0.23±1.07) and executive function performances than *non‐responders* (0.12±0.77 vs‐0.21±0.97), *p*s < .05, but were otherwise comparable. In the intervention arms, the proportion of responders differed significantly: Combined (75%), Physical alone (61%), Active control (44%), (*p* < .05). In the Combined group, *responders* had better global cognition (0.38±0.96 Vs ‐0.20±0.97) and working memory performances (0.25±0.96 Vs ‐0.28±0.47). In the Physical Alone group, *responders* and *non‐responders* performed similarly. In the Active Control group, *responders* had better processing speed performances (0.22±0.31 vs ‐0.33±0.79) than *non‐responders*.

**Conclusion:**

Responders and non‐responders appear to have different baseline cognitive profiles. These results suggest that cognitive and physical training interventions should be tailored and individualized according to baseline cognitive condition.

